# Thermal Transport in Extremely Confined Metallic Nanostructures: TET Characterization

**DOI:** 10.3390/nano13010140

**Published:** 2022-12-27

**Authors:** Huan Lin, Fuhua Shen, Jinbo Xu, Lijun Zhang, Shen Xu, Na Liu, Siyi Luo

**Affiliations:** 1 School of Environmental and Municipal Engineering, Qingdao University of Technology, Qingdao 266033, China; 2 School of Mechanical and Automotive Engineering, Shanghai University of Engineering Science, Shanghai 201620, China

**Keywords:** ultra-thin metallic materials, thermal diffusivity, thermal conductivity, nanostructures, TET

## Abstract

In recent years, the continuous development of electronic chips and the increasing integration of devices have led to extensive research on the thermal properties of ultrathin metallic materials. In particular, accurate characterization of their thermal transport properties has become a research hotspot. In this paper, we review the characterization methods of metallic nanomaterials, focusing on the principles of the transient electrothermal (TET) technique and the differential TET technique. By using the differential TET technique, the thermal conductivity, electrical conductivity, and Lorenz number of extremely confined metallic nanostructures can be characterized with high measurement accuracy. At present, we are limited by the availability of existing coating machines that determine the thickness of the metal films, but this is not due to the measurement technology itself. If a material with a smaller diameter and lower thermal conductivity is used as the substrate, much thinner nanostructures can be characterized.

## 1. Introduction

As an integral part of the semiconductor field, ultra-thin metallic materials are used in solar cells [1], communication [2], aerospace [3], and other applications [4,5,6]. As one of the important criteria for evaluating the performance of different nanomaterials and accurately characterizing the thermal diffusivity of ultrathin metallic materials, thermal transport properties have become an important research direction [7]. However, compared to the electrical transport properties, the characterization of thermal transport within nano-thick metal films is a challenge [8]. 

Wiedemann et al. [9] first discovered that at room temperature, the ratio of electrical conductivity to thermal conductivity was very close for most metals. Later, Lorenz [10] revealed that the ratio was positively correlated with temperature and related to the quantum of electrical charge and the Boltzmann constant. This is the famous Wiedemann–Franz (WF) law, but the WF law is not applicable to nanoscale metal film materials [11,12,13,14,15,16]. Based on the theoretical works related to the optimization of WF law and electrical conductivity [17,18,19,20,21,22,23,24], a series of methods have been developed to experimentally measure the thermal transport properties of metallic nanofilms and metallic nanowires. These methods include the 3ω method [25,26,27,28], the photothermal reflection technique [29,30], the femtosecond laser pumping detection method [31] and the non-stationary electrical heating method [32,33,34,35]. 

A distinctive feature of the 3ω method is that it is universally applicable to a variety of materials [36,37]. However, this method fails in the films thinner than 100 nm, mainly because the thermal contact resistance between the microsensor and the tested film is large and comparable or even larger than the equivalent thermal resistance of the film [38]. Nakamura et al. [39] first measured the thermal diffusivity of 90 nm-thick Pt films on glass substrates at low temperatures from 15 K to 273 K using a post-heating pre-detection type (RF-type) femtosecond thermal reflectometry system. This RF-type system provided the thermal diffusivity of the films at low temperatures, and more information about non-thermal energy transfer processes. Wang et al. [40] studied the heat-transfer mechanism of metal nanofilms under ultrashort pulse laser heating, simultaneously establishing a femtosecond (fs) transient thermal reflection (TTR) technique to measure the transient electron temperature response induced by fs laser heating. They also used a pump-probe technique to ensure the fs time resolution of the experiment. Applying a back heating-front probing mode ensured the electron temperature response, which allowed the authors to determine the electron–phonon coupling coefficient and the propagation velocity of temperature oscillations. Finally, a non-equilibrium thermal diffusion model was employed to fit the temperature profiles of Au films with the thicknesses from 27.2 nm to 55.5 nm.

Laser-based ultrafast time-domain thermal reflection (TDTR) techniques are widely used to measure the thermal conductivity of bulk and thin-film materials [41]. As a pump measurement technique, TDTR requires neither the precisely designed electric heater nor temperature sensor, but only a small amount of sample, which enables one to operate under routine conditions. The frequency-dependent TDTR method is also applicable to thin-film materials. However, their thickness should be greater than the thermal penetration depth through the plane. Liu et al. [42] used this method to determine both the out-of-plane thermal conductivity and the bulk heat capacity for organic–inorganic Zn basin hybrid films with thickness in the range of 40–400 nm.

The photo-thermal technique usually deals with metal samples that act as both the heater and temperature sensor, making the equipment and electrode fabrication process difficult. On the contrary, the electrothermal route is faster and simpler compared with the above methods. Ma et al. [43] measured the in-plane thermal and electrical conductivity of metal nanofilms via direct current heating of suspended films. The advantage of the proposed approach is that contact resistance and thermal resistance can be completely eliminated by integrating the suspended Pt and Au nanofilms with the probe electrodes. Using this method, Boiko et al. [44] determined the thermal and electrical conductivities of 20–60 nm Pt and Au polycrystalline films in the temperature range of 80–300 K. Guo et al. first developed the TET technique to significantly improve the signal electrical frequency. To assess the accuracy of the method, they measured a 25 μm-diameter platinum wire. The thermal diffusivity of the three Pt wires is 2.53 × 10^−5^, 2.54 × 10^−5^, and 2.78 × 10^−5^ m^2^ s^−1^, respectively, which are close to the literature value of 2.51 × 10^−5^ m^2^ s^−1^ (at 300 K). By means of gold coating, the technology can also measure non-conductive nanowires and tubes, but the gold coating needs to be as thin as possible. 

The TET technique is an effective and accurate method (the total uncertainty for thermal diffusivity is 6%) for evaluating the thermal diffusivity of one-dimensional solid materials (including metals and dielectric materials), such as single-walled carbon nanotube bundles [45], graphene materials [46,47,48,49], silkworm silks, [50] silver nanowire network, [51] freestanding micrometer-thick poly films [52], carbon fibers [53,54], etc. In this review, we will focus primarily on the characterization of thermal transport in extremely confined metallic nanostructures using the TET and differential TET technique.

## 2. TET Technique

**Figure 1:**
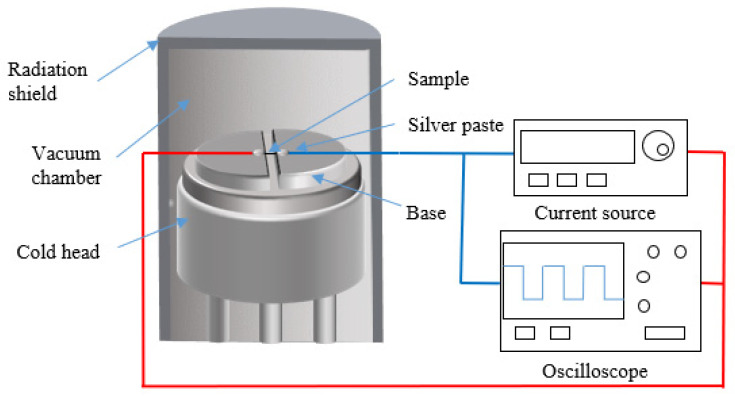
Schematic of the experimental principle and setup for the TET experiment.

The typical experimental setup of the TET technique is shown in Figure 1. During the experiment, both ends of the specimen are suspended between two electrodes. The contact points between the end and the electrode are fixed with a conductive silver glue to increase the electrical and thermal contacts between the sample and the electrodes. The measurement is conducted in a vacuum chamber to eliminate the heat loss through thermal convection.

In the process of the experiment, the step dc current is applied to the material to increase its temperature. The temperature rise in the sample causes the resistance to vary, consequently altering the voltage, which is recorded by an oscilloscope. Since the applied current and the resistance-temperature coefficient of the sample in a narrow temperature range are constant, the temperature change could be derived from the recorded voltage evolution. The thermal diffusivity of the sample is then determined based on the temperature/voltage changing rate.

It is noteworthy that direct measurement is possible only if the material is electrically conductive. Otherwise, the surface of the material is covered with a layer of metal to make it conductive, so the effect of the metallic film should be evaluated and removed.

The length of sample is much larger than its diameter or width and thickness. Therefore, the heat transfer in the samples can be simplified as one-dimensional heat conduction along the length direction. The heat transfer can be described using the equation below [55]:

(1) $$\frac{1}{\alpha }\frac{\partial \theta \left(x,t\right)}{\partial t}=\frac{\partial^{2}\theta \left(x,t\right)}{\partial x^{2}}+\frac{I^{2}R_{0}}{kLA}+\frac{Q}{kLA}$$

where $\theta =T-T_{0}$ $T_{0}$ is the room temperature, *I* is the constant current flowing through the sample, *α* is the thermal diffusivity, *k* is the thermal conductivity, and $R_{0}$ is the electrical resistance before electrical heating. L and A are the length and cross-sectional area of the sample, respectively, and *Q* is the thermal radiation rate. It can be assumed that the electrical heating power per unit volume of the sample is uniform. During Joule heating, the temperature in the sample rises sharply, while the temperature of the electrodes remains constant because of their relatively much larger volume and heat capacity. At the same time, heat flow is transferred from the sample to the electrodes and dissipates from the sample to the surroundings via thermal radiation. Therefore, the boundary conditions are θ(0,t)=θ(L,t)=θ(x,0). The solution to Equation (1) can be obtained by integrating Green’s function. 

The normalized temperature rise (T*) is defined as T*(t) = [*T* (t)-*T*_0_]/ [*T* (t→∞)-*T*_0_], which can be represented as:

(2) $$T^{*}≅\frac{48}{\pi^{4}}\sum_{m=1}^{\infty }\frac{1-{\left(-1\right)}^{m}}{m^{2}}\frac{1-\exp\left[-m^{2}\pi^{2}\alpha_{\mathrm{eff}}t/L^{2}\right]}{m^{2}}$$

where *α*_eff_ is the measured thermal diffusivity. The relationship between the voltage variation recorded by the oscilloscope during the experiment and the mean temperature variation of the sample is as follows:

(3) $$V_{\mathrm{sample}}=IR_{0}+I\eta \frac{4q_{0}L^{2}}{k\pi^{4}}\times \sum_{m=1}^{\infty }\frac{1-{\left(-1\right)}^{m}}{m^{2}}\frac{1-\exp\left[-m^{2}\pi^{2}\alpha_{\mathrm{eff}}t/L^{2}\right]}{m^{2}}$$

where *η* is the temperature resistivity coefficient and *q*_0_ is the electrical power per unit volume. 

The normalized temperature rise ($T_{}^{*}$) is calculated from the experimental data as $T^{*}=\left(V_{\mathrm{sample}}-V_{0}\right)/\left(V_{1}-V_{0}\right)$, where *V*_0_ and *V*_1_ are the initial and steady-state voltages of the sample. After obtaining $T_{}^{*}$, different values of *α*_eff_ are used to fit the experimental results $T_{}^{*}$ based on Equation (2). According to the least squares fitting technique, the value giving the best fit of $T_{}^{*}$ is used as the *α*_eff_ of the sample.

For the non-conductive materials, the value of *α*_eff_ includes thermal radiation and metal coating effects. Thus, it can be written as [56,57,58]:

(4) $$\alpha_{s}=\alpha_{\mathrm{eff}}-\frac{1}{\rho c_{p}}\frac{16\varepsilon_{r}\sigma T_{0}^{3}}{D}\frac{L^{2}}{\pi^{2}}-\frac{L_{\mathrm{Lorenz}}T_{\mathrm{ave}}L}{RA\rho c_{p}}$$

where α_s_ is the thermal diffusivity of the substrate, D is the diameter of the sample to be measured, ε_r_ is the surface emissivity, σ=5.67 × 10^−8^ W·m^−2^·K^−4^ is the Stefan–Boltzmann constant, and *ρc_p_* is the volumetric specific heat of the material. *L*_Lorenz_ is the Lorenz number, *T*_ave_ and *R* are the average temperature and resistance of the sample during the TET. The second term on the right side of the equation is the thermal radiation effect, and the third term refers to the coating effect. The radiation effect can be taken out by linearly fitting the *α_eff_*–*L*^2^/*D* curve to *L*^2^/*D* = 0. The slope of the fitting line is $16\varepsilon_{r}\sigma T_{0}{}^{3}/(\pi^{2}\rho c_{p})$. As the other parameters are all known, the emissivity of samples can be calculated from the slope of the curve. If the material is electrically conductive, it requires no metal coating; in this case, only the radiation impact should be considered. 

## 3. Differential TET Technique 

Since the independent structure of nanometer-thick materials is relatively weak to suspend, the differential TET technique [58,59,60] was developed to measure the in-plane thermal transport of metallic nanostructures so as to accurately represent their electrical conductivity, thermal conductivity, and Lorenz number. 

**Figure 2:**
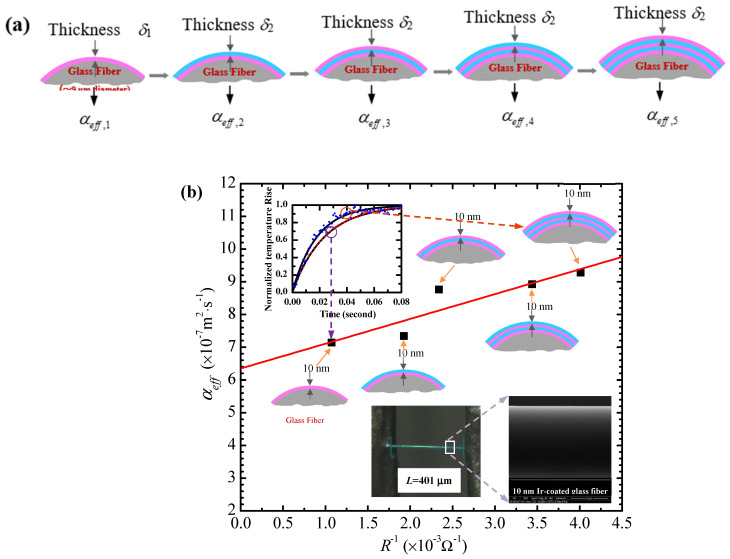

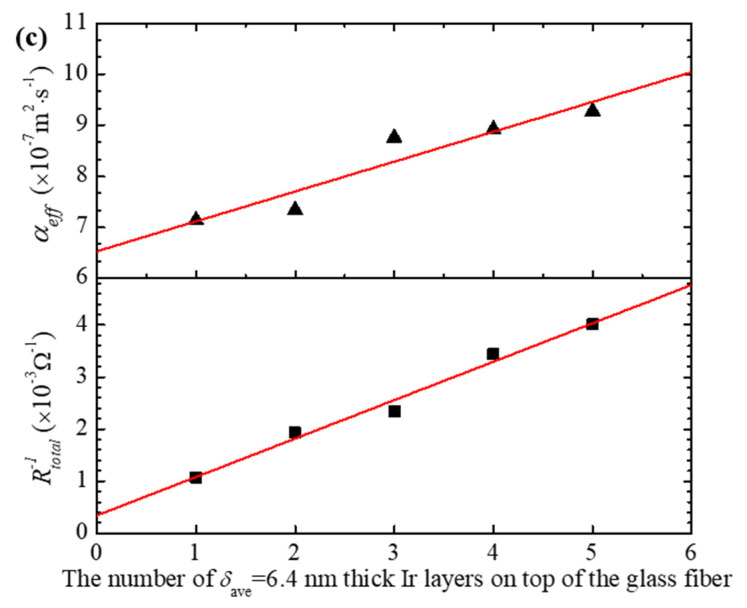
(**a**) Schematic cross-section of a substrate coated with different layers of nanofilms. The effective thermal diffusivity variation against the amount of metallic coating layers and electrical conductance (R^−1^) used to obtain the Lorenz number, thermal conductivity, and electrical conductivity. (**b**) The effective thermal diffusivity versus the inverse electrical resistance of a substrate coated with 6.4 nm-thick Ir layers. (**c**) Linear fitting curves of the effective thermal diffusivity and resistance change with the number of Ir layers on the substrate. (Reprinted with permission from Ref. [56] Copyright John Wiley and Sons Small.).

Since the low-dimensional materials possess low thermal conductivity, they can be used as the substrates to brace the ultrathin films during testing. As shown in Figure 2a,b, a metallic layer is applied in the TET experiment to measure *α*_eff_. It is clear from Equation (4) that *α*_eff_ is influenced by three factors, which are *α*_s_, the thermal radiation effect, and the coating effect. Among them, *α*_s_ is a constant and the thermal radiation influence remains generally unchanged and can be neglected. If the coating is added, the value *α*_eff_ will be changed accordingly. Therefore, the relation between *α*_eff_ and the number of layers can be established. The effective thermal diffusivity of the sample has an expression as [56] $\alpha_{\mathrm{eff}}=\alpha_{s}+\frac{4\cdot n\cdot \delta_{\max}}{\pi D{\left(\rho c_{p}\right)}_{s}}\left[k_{c}-\alpha_{s}{\left(\rho c_{p}\right)}_{c}\right]$, where *α*_s_ is the thermal diffusivity of the substrate, being a constant value. The subscript *c* indicates the metallic structure. As shown in Figure 2c, *α*_eff_ changes with *n* conforming to a linear law, and its slope can be obtained from the fitting. Therefore, the inherent thermal conductivity of a thin coating structure can be accurately obtained according to the theoretical model. Using the same method, the electrical conductivity and Lorenz number can also be determined.

Lin et al. measured *k* of 6.4 nm-thick gold films [61] and 7 nm-to-subnanometrically thick Ir films [56,60] by applying the differential TET technique. The average thermal conductivity of Ir films deposited on glass fibers was reduced by 51.2% compared with the bulk value (147 W·m^−1^·K^−1^) at 311 K. Moreover, the decrease in electrical conductivity was much faster than in thermal conductivity, which caused the Lorenz number to increase to 6–8 × 10^−8^ W Ω K ^−2^. It was noted that the thermal conductivity of the Au film on silkworm silks was 50% of that on glass fibers. However, the thermal conductivity of the 6.4 nm-thick Ir film on silkworm silks was only slightly higher than that on the glass fiber. These variations in thermal conductivity are probably caused by the difference between the film structures; that is, Ir film has a finer crystalline size than that of Au.

Dong et al. characterized the thermal and electronic transport properties of 3.2 nm gold films applied onto the alginate fibers via the differential TET technique [62]. It was concluded that the thermal and electrical conductivity were significantly reduced by 76.2% and 93.9%, respectively, compared to the corresponding values of the bulk material. Meanwhile, the calculated Lorenz number was almost three times higher than the Lorenz number of the bulk material.

The thermal and electrical conductivity of the metallic structures deposited on the substrates are lower than those of the bulk material. Additionally, the substrate structure exerts an important impact on the electrical and thermal properties of the metallic structure. For instance, the silkworm silk has lower thermal conductivity, and the electron tunneling along with hopping in this type of fiber can improve the electron conductivity of the metallic structure. Therefore, the silkworm silk is more suitable as a substrate material in flexible electronic devices.

**Figure 3:**
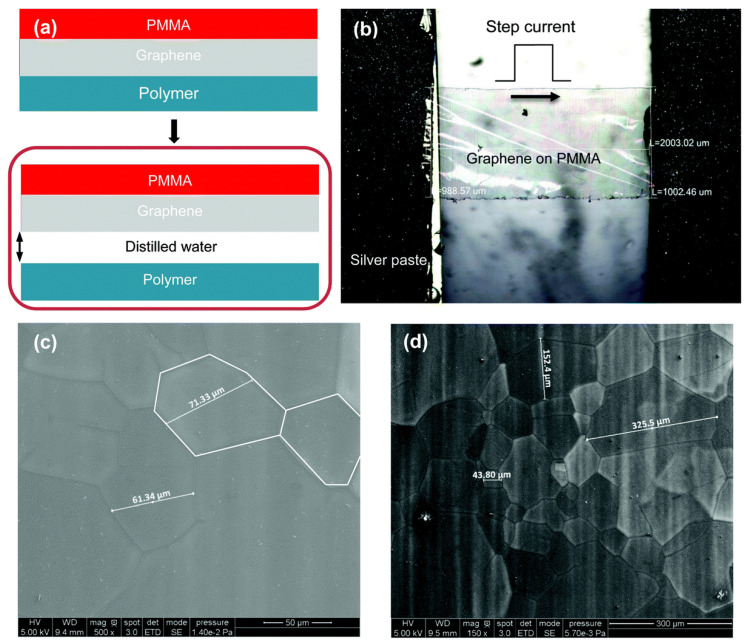
(**a**) Procedures for acquiring the sample of desired size from graphene. (**b**) Microscopic image of the graphene sample between the electrodes. (**c**,**d**) SEM images of the sample. The characteristic size of grains can be clearly seen in the range of tens to hundreds of microns. (Reproduced from Ref. [63] with permission from The Royal Society of Chemistry).

Liu et al. [63] measured *k* of the chemical vapor deposited (CVD) graphene supported on poly(methyl methacrylate) (PMMA) using the differential TET technique shown in Figure 3. *k* of 1.33-layered, l.53-layered, 2.74-layered, and 5.2-layered supported graphene were 365 W·m^−1^·K^−1^, 359 W·m^−1^·K^−1^, 273 W·m^−1^·K^−1^, and 33.5 W·m^−1^·K^−1^, respectively. These values were, on average, eight times lower than those reported for suspended graphene (*k* = 3000 W·m^−1^·K^−1^). The reduction in *k* was due to the suppression of ZA phonons by the substrate. The abundant C atoms in PMMA were more readily coupled with graphene than other atomic substrates. Hence, the differential TET technique is a fast and reliable method used to measure *k* of graphene. This work shows that the differential TET technique has great potential for future research on the thermal properties of graphene.

## 4. Summary and Prospects

In summary, the differential TET technique is one of the most optimal techniques for characterizing thermal transport properties in extremely confined metallic nanostructures, allowing one to precisely determine their thermal conductivity, electrical conductivity, and Lorenz number. Moreover, it possesses significant advantages over other widely used methods in terms of implementation simplicity, high signal-to-noise ratio, and high measurement accuracy. The disadvantages of the TET technique are that it cannot measure samples with extremely low resistance (less than 1 ohm) and measurement needs to be performed in a vacuum environment. The surface radiation effect cannot be ignored if the sample has a very large aspect ratio (*L*/*D*).

At present, the coating thickness can be explicitly controlled to the order of 0.1 nm. Additionally, we are limited by the availability of existing coating machines that determine the thickness of the metal films, but this is not due to the measurement technology itself. If the thermal conductivity of a substrate material is extremely low, the heat transfer between the substrate and the metal coating can be effectively reduced. Therefore, the substrates with low thermal conductivity and small diameter will soon make it possible to use very thin metallic structures. The TET technique will provide powerful aid in mastering the intrinsic heat transport properties of new materials, and it will be helpful for the development of new materials.

## Data Availability

The data presented in this study are available on request from the corresponding author.
